# The embryonic type of *SPP1* transcriptional regulation is re-activated in glioblastoma

**DOI:** 10.18632/oncotarget.14092

**Published:** 2016-12-22

**Authors:** Magdalena Kijewska, Marta Kocyk, Michal Kloss, Karolina Stepniak, Zbigniew Korwek, Renata Polakowska, Michal Dabrowski, Anna Gieryng, Bartosz Wojtas, Iwona A Ciechomska, Bozena Kaminska

**Affiliations:** ^1^ Laboratory Molecular Neurobiology, The Nencki Institute of Experimental Biology of the Polish Academy of Sciences; ^2^ Laboratory Molecular Bases of Aging, The Nencki Institute of Experimental Biology of Polish Academy of Sciences, Warsaw, Poland; ^3^ Jean-Pierre Aubert Research Center, INSERM U837, Lille, France

**Keywords:** osteopontin, glioma initiating cells, transcription factors, stemness factors, self-renewal

## Abstract

Osteopontin (SPP1, a secreted phosphoprotein 1) is primarily involved in immune responses, tissue remodelling and biomineralization. However, it is also overexpressed in many cancers and regulates tumour progression by increasing migration, invasion and cancer stem cell self-renewal. Mechanisms of *SPP1* overexpression in gliomas are poorly understood. We demonstrate overexpression of two out of five *SPP1* isoforms in glioblastoma (GBM) and differential isoform expression in glioma cell lines. Up-regulated *SPP1* expression is associated with binding of the GLI1 transcription factor to the promoter and OCT4 (octamer-binding transcription factor 4) to the first *SPP1* intron of the *SPP1* gene in human glioma cells but not in non-transformed astrocytes. GLI1 knockdown reduced *SPP1* mRNA and protein levels in glioma cells. GLI1 and OCT4 are known regulators of stem cell pluripotency. GBMs contain rare cells that express stem cell markers and display ability to self-renew. We reveal that *SPP1* is overexpressed in glioma initiating cells defined by high rhodamine 123 efflux, sphere forming capacity and stemness marker expression. Forced differentiation of human glioma spheres reduced *SPP1* expression. Knockdown of *SPP1*, *GLI1* or *CD44* with siRNAs diminished sphere formation. C6 glioma cells stably depleted of Spp1 displayed reduced sphere forming capacity and downregulated stemness marker expression. Overexpression of the wild type Spp1, but not Spp1 lacking a Cd44 binding domain, rescued cell ability to form spheres. Our findings show re-activation of the embryonic-type transcriptional regulation of *SPP1* in malignant gliomas and point to the importance of SPP1-CD44 interactions in self-renewal and pluripotency glioma initiating cells.

## INTRODUCTION

Osteopontin/SPP1, a secreted phosphoprotein 1, acts via integrin receptors and the glycoprotein CD44, and regulates adhesion, migration, invasion, chemotaxis and cell survival [1]. Multi-functionality of osteopontin is due to the presence of different isoforms, generated by an alternative splicing, and various post-translational modifications such as phosphorylation, sulfation, glycosylation and protelytic cleavage. The levels of *SPP1* mRNA are up-regulated in many malignant cancer tissues, and elevated levels of SPP1 in patients’ tumour tissue and blood are associated with poor prognosis [2, 3]. SPP1 modulates many functions of cancer cells: it stimulates cancer cell proliferation and invasion, and supports tumour angiogenesis [4, 5] and distant tumour outgrowth by instigating dormant tumours [6]. On the other hand, SPP1 expression is increased under acute and chronic inflammatory conditions, wound repair and fibrosis. SPP1 is implicated in chemotaxis and recruitment of immune cells to inflamed sites, and production of inflammation mediators by immune cells [7, 8]. These various and to some extent opposing functions of SPP1 are attributed to its differential posttranscriptional processing in normal and transformed cells [9–11].

Glioblastoma (GBM) is the most common primary brain tumour in adults and its treatment remains a major challenge for clinicians because these aggressive and invasive tumours are highly resistant to radio- and chemotherapy [12]. Previous studies reported the elevated expression of three *SPP1* isoforms in tumour tissues and sera from GBM patients, and found an inverse correlation of its expression with patient survival [13–16]. Isoforms of *SPP1* displayed different effectiveness in stimulation of glioma invasion and cell survival [17]. GBM contains a subpopulation of glioma initiating cells (GIC) with stem cell features and an ability to self-renew. These cells are believed to contribute to therapy resistance and tumour recurrence [18, 19]. A couple of recent studies demonstrated the important role of autocrine and paracrine SPP1-CD44 signalling in maintenance of glioma initiating cells [20, 21].

Despite numerous reports regarding SPP1 up-regulation in many cancers, there is a scarce information regarding the transcriptional regulation of *SPP1*, in particular in cancer stem cells. The *SPP1* expression is regulated mainly at the level of transcription [22]. Deletion analyses of the *SPP1* gene promoter and gel shift studies demonstrated c-Myc and OCT-1 binding to the proximal promoter of *SPP1* gene in U251MG and U87MG human glioma cells [22]. Transcription factors ETS-1 and RUNX2 regulated *SPP1* expression in colorectal cancer cells [23]. In melanoma cells transcription factors c-Myb [24], AML-1a and C/EBPα bind to the *SPP1* gene promoter [25] and the transcription factor GLI1, a mediator of Hedgehog (HH) signalling have been shown to regulate SPP1 expression [26]. Transcriptional regulation of *SPP1* in GBM cells and its role in GIC compartment needs further clarification.

In this study we present the expression pattern of five *SPP1* isoforms in low and high grade gliomas, five glioma cell lines and non-transformed astrocytes, and transcriptional regulation of *SPP1* by stemness transcription factors GLI1 and OCT4, expressed in glioblastoma cells, but not in normal astrocytes. Moreover, we report up-regulation of the *SPP1* expression in glioma initiating cells, defined by high efflux capacities, sphere forming abilities and the upregulated expression of stemness markers. In glioma sphere cultures undergoing forced differentiation the expression of *SPP1* was reduced. Using siRNA and shRNA-mediated gene interference we demonstrated the involvement of SPP1/osteopontin in glioma sphere formation and the importance of SPP1-CD44 interactions.

## RESULTS

### Differential expression of *SPP1* isoforms in low and high grade gliomas and human glioma cell lines

**Figure 1:**
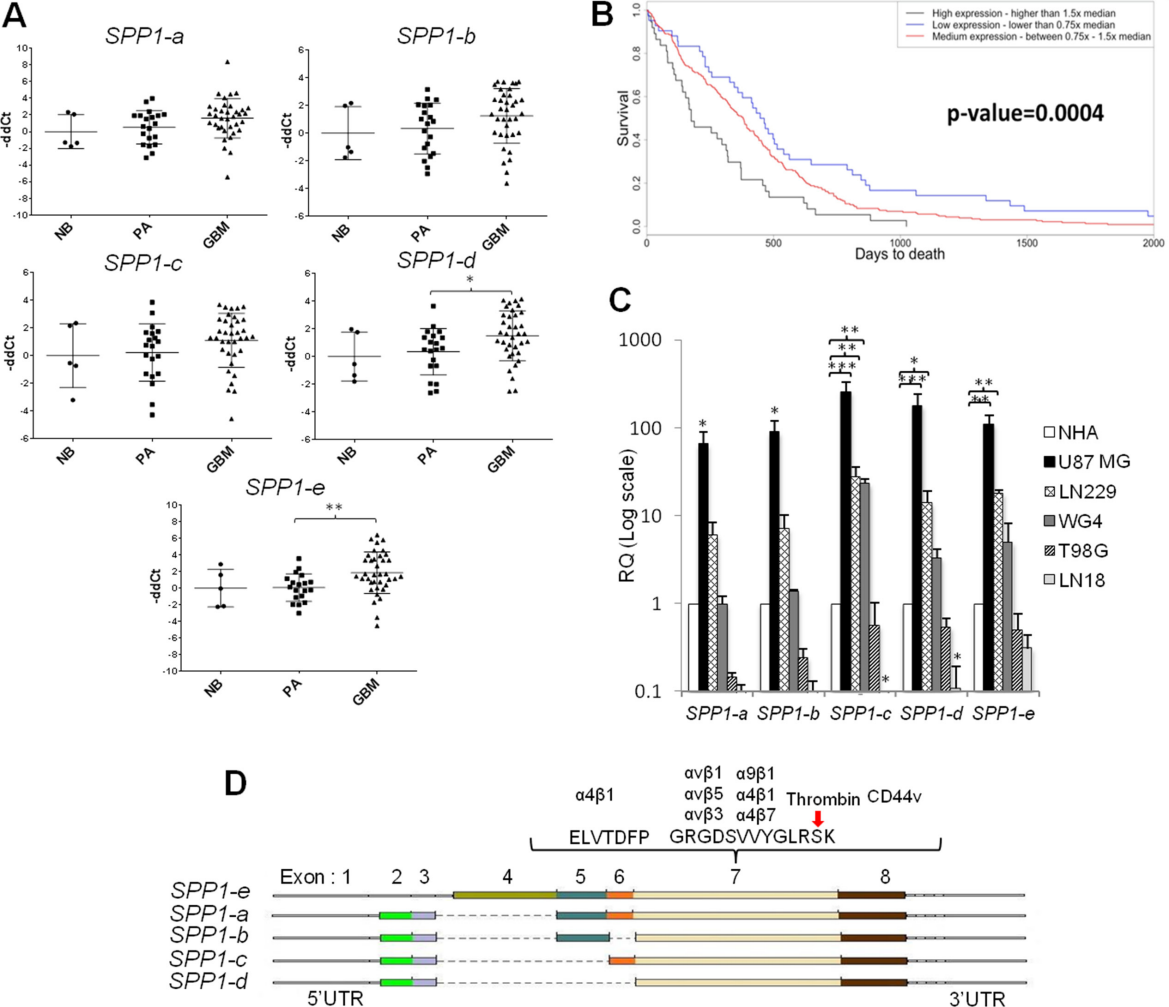
The expression pattern of *SPP1* splicing variants in glioma clinical samples and human glioma cell lines (**A**) *SPP1–e* and *SPP1-d* isoform mRNAs are up-regulated in GBM. The expression level of five *SPP1* isoforms was assessed in 36 glioblastoma (GBM), 20 pilocytic astrocytoma (PA) and 5 normal brains (NB) using qPCR. Data were normalized to the expression of *GAPDH* mRNA determined in the same sample. *T-test* analysis was performed on -ddCt results, *P* values were considered significant when *P ≤ 0.05 and **P ≤ 0.01. (**B**) Prognostic value of the *SPP1* expression in GBMs (WHO grade IV) in the TCGA cohort. Kaplan-Meier plots were estimated according to different *SPP1* gene expression and overall survival of all GBM patients (*n* = 359). A chi-square test was used to evaluate differences in survival of patients with *SPP1* expression lower or higher than median. (**C**) Relative expression of *SPP1* splicing variants in human glioma cell lines versus non-transformed astrocytes. Human T98G, LN18, LN229, U87 MG, GBM patient derived WG4 glioma cells, and normal human astrocytes (NHA, Lonza) were used. Data were normalized to the expression of *GAPDH* mRNA and represent mean ± s.d., of three independent passages. *T-test* analysis was performed on -ddCt results, *P* values were considered significant when *P ≤ 0.05, **P ≤ 0.01 and *** for *P ≤* 0.001. (**D**) A scheme shows the organisation of the *SPP1* gene in different isoforms and location of the most important functional domains.

Previous studies have determined the expression of *SPP1-a,-b,-c* isoforms in tumour tissues [2, 3]. Examination of *SPP1* records in the NCBI database (http://www.ncbi.nlm.nih.gov/gene/6696) shows the presence of five isoforms of this gene. We determined their expression in normal brains (*n* = 5, one being a mixture of 24 RNA samples), benign juvenile pilocytic astrocytomas (PA, WHO grade I, *n* = 20) and highly malignant glioblastomas (GBM, WHO grade IV, *n* = 57). *SPP1-e (*former *SPP1-a)* and *SPP1-d* isoforms were significantly overexpressed in GBMs (Figure 1A). The analysis of TCGA (The Cancer Genome Atlas) data set confirmed these results. Kaplan-Meyer survival plot derived from TCGA data illustrates negative correlation between high *SPP1* expression and survival time (Figure 1B). The expression of *SPP1* did not differ significantly between four GBM subtypes (Supplementary Figure S1). A distinctive expression pattern of *SPP1* isoforms was detected in a panel of human glioma cells when compared to normal astrocytes (NHA). The expression of all *SPP1* isoforms was increased in U87MG glioma cells, *SPP1-c, -d, -e* mRNA levels were upregulated in LN229 glioma cells; GBM patient-derived WG4 cells had the higher *SPP1-c* level in comparison to non-transformed human astrocytes. T98G and LN18 glioblastoma cells had lower levels of *SPP1* mRNAs than astrocytes (Figure 1C). A scheme in the Figure 1D depicts exons and corresponding protein domains present in various SPP1 isoforms as well as sites of potential interactions with other proteins. The *SPP1-e* mRNA encodes a full length protein, while *SPP1-d* mRNA encodes the *SPP1* isoform lacking 4, 5 and 6 exons.

### Transcription factors GLI1 and OCT4 are involved in transcriptional control of *SPP1* expression in glioma cells

**Figure 2:**
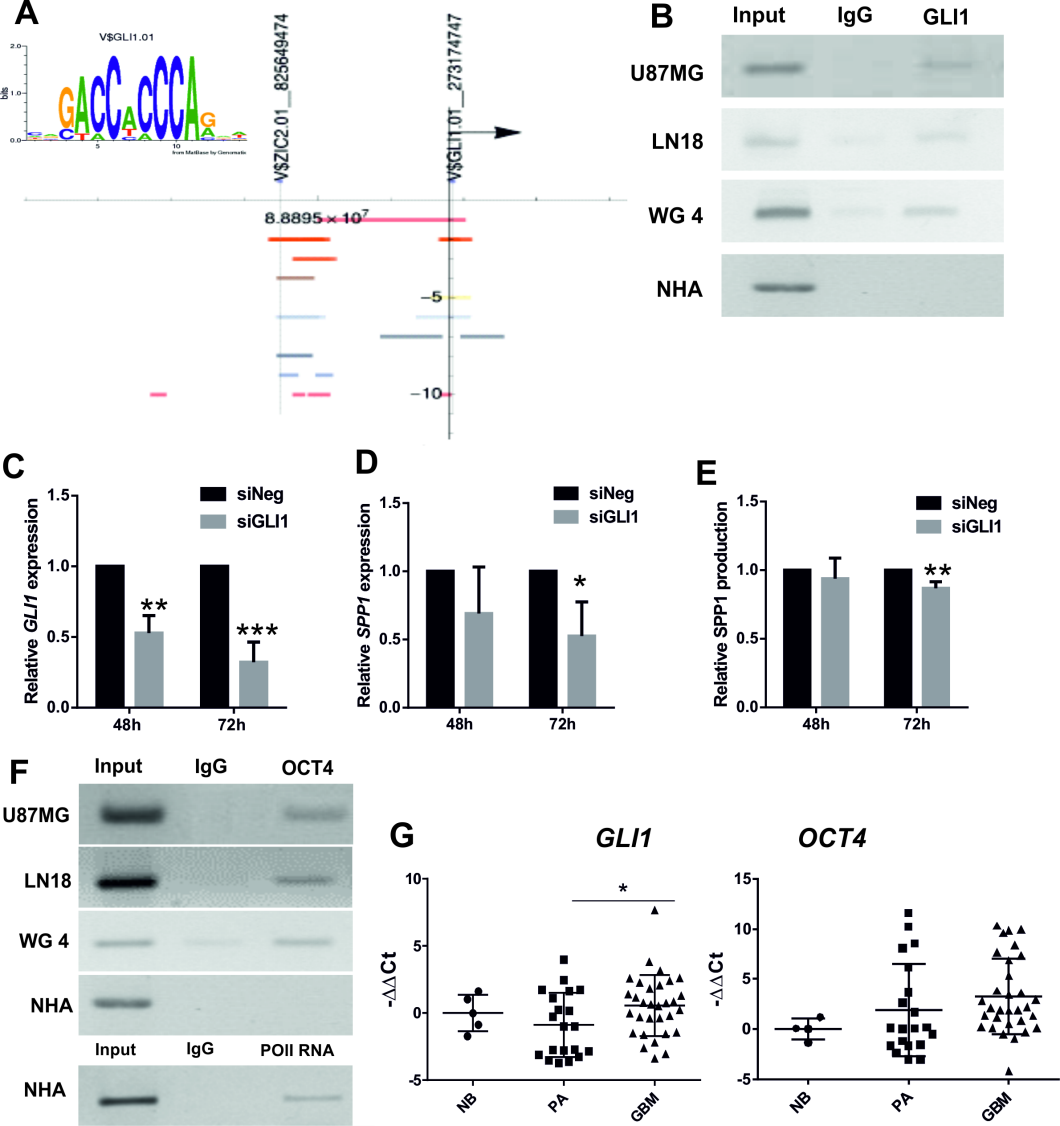
GLI1 and OCT4 participate in transcriptional regulation of *SPP1* expression in glioma cells (**A**) The GLI1 binding sequence logo was generated by MathInspector and the results of computational analysis of the human *SPP1* gene promoter revealed the presence of two potential GLI1 binding sites. Coloured bands represent positions of the regulatory sites (open chromatin, histone modifications) and the putative GLI1 binding sites with their chromosomal locations identified by MathInspector and the Ensembl funcgen database as described [27]; an arrowhead shows direction of transcription. (**B**) Binding of GLI1 to the *SPP1* gene promoter was detected by chromatin immunoprecipitation (ChIP) in three human glioma cell lines, but not in non-transformed human astrocytes (NHA); an input represents a positive control; IgG is a neutral antibody. (**C**) Knockdown of *GLI1* expression in U87MG glioma cells after siRNA transfection. Human GLI1 was knocked-down using ON-Target PlusSMARTpool Human GLI1 (Dharmacon) with ON-TARGETplus Non-targeting Pool as a control. The results are expressed as mean ± s.d.; *P* values ≤ 0.05 were considered significant; *n* = 3. (**D**, **E**) Knockdown of *GLI1* expression in U87MG glioma cells decreased the *SPP1* mRNA level and reduced the level of secreted osteopontin. The results are expressed as mean ± s.d.; *P* values ≤ 0.05 were considered significant, *n* = 4. (**F**) The OCT4 transcription factor binds to the first intron of the *SPP1* gene in three glioma cell lines but not in NHA (IgG is a neutral antibody). Primers for the potential OCT4 binding site in the first intron of the *SPP1* gene corresponded to the Oct4 binding site in the murine *spp1* gene previously described [28]. ChIP with anti-Pol II antibody and binding to the *GAPDH* gene promoter was performed as a positive control of ChIP reaction in NHA. The PCR products were resolved in 1% agarose gel with ethidium bromide and visualized with UV light. (**G**) The expression of *GLI1* and *OCT4* was assessed in 36 glioblastoma (GBM), 20 pilocytic astrocytoma (PA) and 5 normal brains (NB) using qPCR. Data were normalized to the expression of *GAPDH* mRNA determined in the same sample. *T-test* analysis was performed on -ddCt results, *P* values were considered significant when *P ≤ 0.05.

We performed a computational analysis of the human *SPP1* promoter region using the Nencki Genomics Database [27] which integrates information about sequence motifs and epigenetic modifications in the non-coding regions, conserved between human, rat and mouse. Two putative GLI1 binding sites were identified in the human *SPP1* gene promoter (Figure 2A). GLI1 (Glioma-Associated Oncogene Homolog 1) is a transcription factor activated downstream Hedgehog signalling pathway. We demonstrated binding of GLI1 to the proximal promoter of *SPP1* gene by chromatin immunoprecipitation (ChIP) assay in three human glioma cell lines, including primary human WG4 glioma cultures, but not in NHA (Figure 2B). Knockdown of *GLI1* expression in glioma cells (Figure 2C) significantly reduced the levels of *SPP1* mRNA and protein production (Figure 2D–2E).

A stemness factor Oct4 was shown to bind within the first intron of the murine *Spp1* gene [28] and regulate its expression in murine embryonic cells. Using chromatin immunoprecipitation we found binding of the OCT4 protein to the first intron of the *SPP1* gene in human glioma cells, but not in NHA (Figure 2F). Effective binding of RNA Polymerase II to the constitutive promoter was assessed as a positive control to ensure a good quality of the immunoprecipitated DNA from NHA (Figure 2F). We have attempted silencing of the *OCT4* expression with the specific siRNA, however, due to barely detectable OCT4 levels in glioma cells, we could not verify its knockdown by Western blotting.

The levels of *GLI1* and *OCT4* mRNAs were determined in the same samples of normal brain, pilocytic astrocytomas and glioblastomas analyzed for *SPP1* expression. The *GLI1* mRNA level was significantly up-regulated in GBMs in comparison to low grade tumors and normal brains. The *OCT4* expression was detectable in all tested tumors and was not significantly different in GBMs versus PAs (Figure 2G). The presented results show co-expression and binding of GLI1 and OCT4 transcription factors to the *SPP1* gene. The transcriptional regulation of the *SPP1* expression by GLI1 (and likely OCT4) in glioma cells resembles the stem cell-type regulation.

### *SPP1* expression is up-regulated in glioma initiating cells

**Figure 3:**
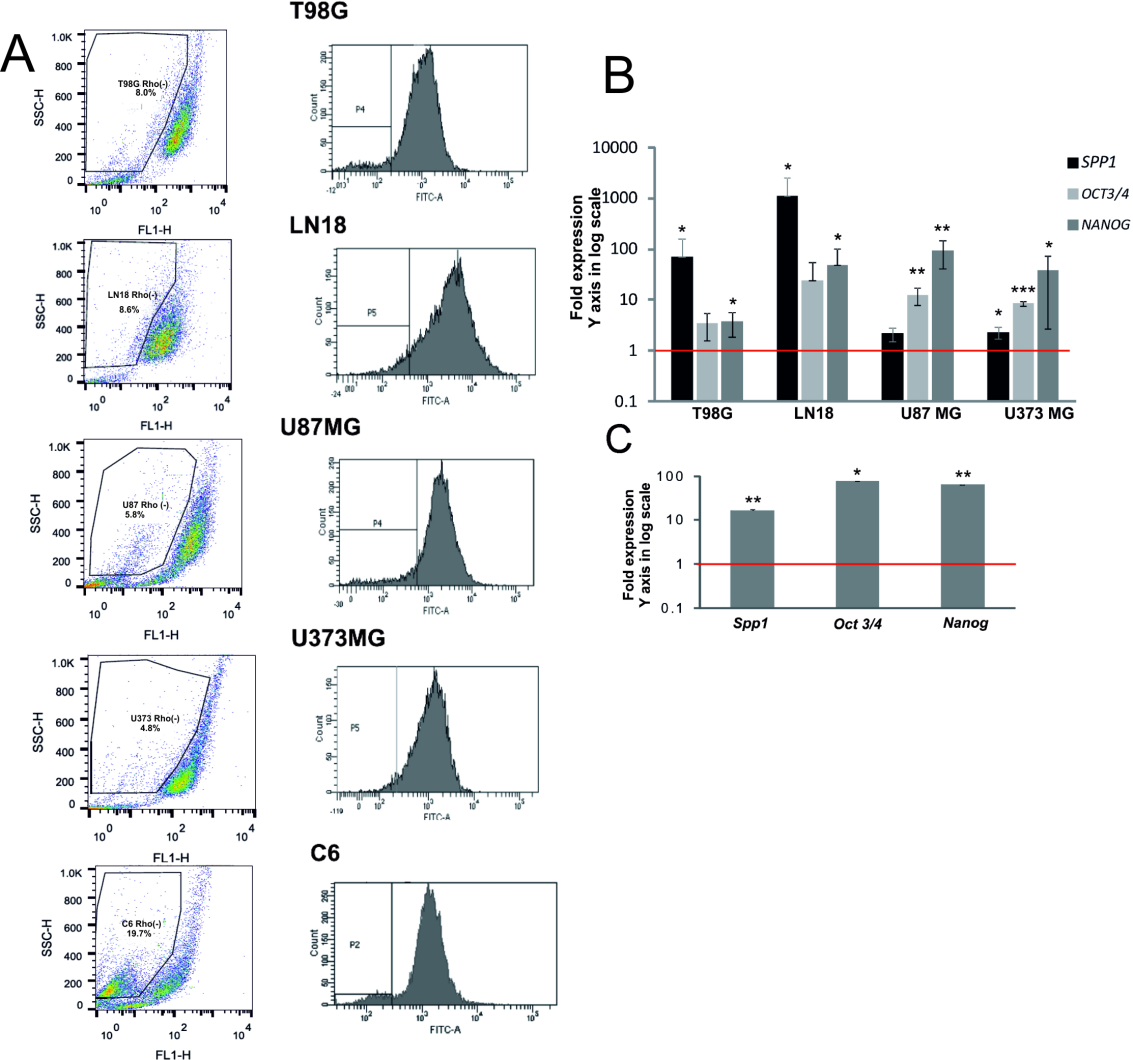
*SPP1* expression is up-regulated in glioma initiating cells (**A**) Representative examples of dot plots and histograms of Rhod 123(–) subpopulations from 5 glioma cell lines were acquired by flow cytometry. Glioma cells (1 × 10^7^) were trypsinized and incubated with 0.1 μg/ml Rhod123 for 20 min in 37°C. After washing the cells were placed in 37°C for 90 min for Rhod123 exclusion in a dark compartment. Cells kept on ice to inhibit exclusion of Rhod123 were used as a positive control for gating in flow cytometry. Fractions of Rhod123(+) and Rhod123(–) cells were sorted using FACS Aria and a left panel shows a gating strategy. (**B**) Analysis of *SPP1, OCT3/4* and *NANOG* gene expression in Rhod 123(–) subpopulations sorted from four human glioma cell lines. Total RNA was isolated from sorted cells using Qiagen RNeasy kit and the levels of *SPP1, NANOG* and *OCT3/4* mRNA were determined by qPCR in Rhod 123(+) and Rhod 123(–) subpopulations; their expression in Rhod 123(+) subpopulations was taken as 1 (a red line). *P* values were calculated with a *t-test* and considered significant when *P ≤ 0.05 and **P ≤ 0.01. (**C**) Quantification of the expression of *Spp1, Oct3/4* and *Nanog* in Rhod 123(–) subpopulations isolated from rat C6 glioma cells versus their levels in Rhod 123(+) subpopulations taken as 1 (a red line); *n* = 3.

Transcription factors GLI1 and OCT4 are involved in a control of self-renewal of both somatic stem cells and cancer stem cells [29]. The observed regulation of *SPP1* expression by stemness transcription factors led us to quantify the *SPP1* mRNA levels (specifically *SPP1-e*, a full form of *SPP1)* in a subpopulation enriched in glioma initiating cells (GIC). These cells are implicated in tumour formation and recurrence [30, 31]. To obtain a subpopulation enriched in GIC, we isolated a side population (SP) using a Rhodamine123 (Rhod123) exclusion assay and flow cytometry. Stem-like cells exclude dyes more efficiently due to up-regulated expression/activity of ABC transporters [32]. Gating strategy of the Rhod123(–) subpopulation is presented in the Supplementary Figure S2. The lower panel shows gating of the Rhod123(–) subpopulation stained with propidium iodide to control for cell viability and demonstrates a clear separation of those cells from Rhod123(+) and dead cells. It shows that FACS sorted Rhod123(–) cells were alive and excluded a Rhodamine123 dye more efficiently. Representative dot plots and histograms of sorted cells are presented in Figure 3A. The Rhod123(–) subpopulation was detected in all tested cell lines and constituted from 3% to 8% of the total cell population in agreement with previous reports [33]. The percentage of Rhod123(–) cells was: 8.1 ± 7.9% in T98G cells, 4.46 ± 3.56% in LN18 cells, 6.85 ± 1.05% in U87MG cells, 4.3 ± 2.9% in U373MG cells and 18.65 ± 7.39% in C6 cells.

The expression of *NANOG* and *OCT3/4* (coding for stemness markers) in sorted Rhod123(–) subpopulation and bulk cells was measured by a quantitative PCR and considered as an indicator of GIC enrichment [34]. The levels of *NANOG* and *OCT3/4* mRNA were significantly higher in sorted Rhod123(–) cells than in the bulk cells in all tested glioma cultures (Figure 3B–3C). The *SPP1* expression was 1000-fold up-regulated in the Rhod123(–) LN18 cells. The relative *SPP1* overexpression in the Rhod123(–) subpopulation of T98G and LN18 cells versus bulk cells was similar in magnitude to *SPP1* overexpression in U87MG and U373MG cells (having the highest *SPP1* expression in the total cell population) when compared to NHA. The levels of *Spp1*, *Nanog* and *Oct3/4* mRNAs were augmented in the Rhod123(–) cells isolated from the rat C6 glioma cultures when compared with their expression in non-transformed astrocytes (Figure 3C).

**Figure 4:**
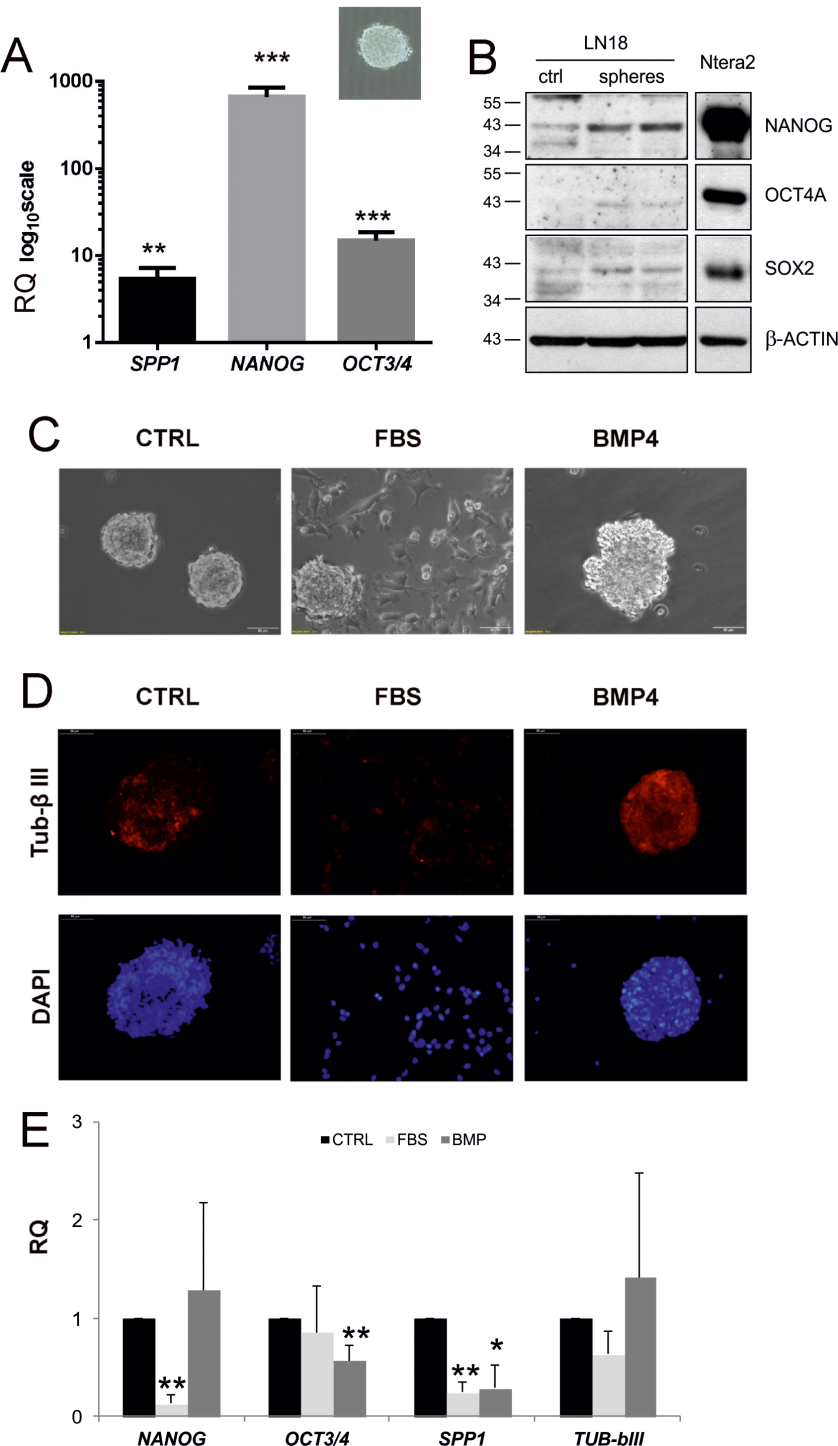
*SPP1* expression is up-regulated in glioma spheres and reduced after forced differentiation (**A**) Human LN18 glioma spheres express the higher levels of *SPP1, OCT3/4* and *NANOG* than adherent cells. For sphere forming assay, cells were seeded at a low density (4000 cells/ml) onto non-adherent plates and cultured in DMEM/F-12 medium, supplemented with B27, 20 ng/ml bFGF, 20 ng/ml EGF and antibiotics; inset shows a representative sphere, 40× magnification. After 14 days resulting spheres and adherent cultures were collected by centrifugation and lysed in Qiagen RLT lysis buffer. Gene expression was determined with qPCR; data are presented as means ± s.d. *T-test* analysis was performed, *P* values were considered significant when *P ≤ 0.05 and **P ≤ 0.01; *n* = 3. (**B**) Western blot analysis of stemness factors NANOG, OCT4A, SOX2 in adherent (ctrl) and LN18 spheres grown for 14 days. The expression of stemness markers at the protein level was additionally evaluated in human embryonic teratoma Ntera2 cells expressing those stemness markers at very high levels. Samples were run on the same blots to determine the correct band size of proteins. (**C**) Spheres cultured for 8 days were differentiated for 5 days in the presence of 100 ng/ml BMP4 or 2% FBS. Light microscopy shows morphological alterations of LN18 sphere cultures, in particular spreading of cells and attachment to the bottom of plates when spheres were cultured in the medium containing 2% FBS. (**D**) Representative images of cells stained for TUB-β III (a neuronal marker) and co-stained with DAPI to visualize cell nuclei. (**E**) Addition of BMP4 and 2% serum resulted in reduction of the expression of *SPP1* in LN18 cells. Spheres cultured for 8 days were differentiated for 5 days in the presence of 100 ng/ml BMP4 or 2% FBS. BMP4 treatment reduced significantly *NANOG* expression, while serum addition resulted in the reduction of *OCT3/4* mRNA level. Data are means ± s.d., *n* = 5.

Cancer stem cells could be grown and maintained in suspension using the neurosphere assay. These 3-D sphere cultures formed by clonally growing, anchorage-independent cells in serum-free media supplemented with specific growth factors are employed to enrich in self-renewing, multipotent cells. The resulting spheres can be expanded or forced to differentiate into cells expressing neuronal and glial differentiated cell markers [19]. It has been demonstrated that cells growing as glioma sphere cultures are able to form tumours closely resembling human pathology when transplanted intracerebrally to immunosuppressed mice [35]. To determine whether *SPP1* is overexpressed in GIC, we measured *SPP1* mRNA levels in adherent and 3-D, sphere cultures developed from human and rat glioma cells. We found the increased expression of *NANOG, OCT3/4* and *SPP1* mRNAs in LN18 spheres (Figure 4A) and C6 spheres (not shown). Western blot analysis confirmed the higher expression of stemness markers: NANOG, OCT4A and SOX2 in LN18 spheres when compared with parental flat cultures (Figure 4B). The expression of those markers in Ntera2 cells (human embryonic teratoma cells expressing stemness markers at very high levels) served as a positive control (Figure 4B).

### The expression of *SPP1* is decreased upon GIC differentiation

Serum and bone morphogenetic proteins (BMP) are potent inducers of glioma differentiation *in vitro* and *in vivo*, and have been shown to reduce a number of glioma initiating cells, decrease glioma proliferation and induce astrocytic or neuronal-like differentiation, respectively [19]. We demonstrate that LN18 spheres undergo differentiation in the presence of 2% FBS or 100 ng/ml BMP4 (Figure 4C). Light microscopy shows morphological alterations of LN18 spheres: 5 days after treatments the cells migrated out of spheres and became flatten and adherent, in particular in the presence of 2% FBS (Figure 4C). Immunoflurescence analysis of spheres exposed to BMP4 shows an increase of the TUB-βIII staining (Figure 4D), which is consistent with neuron-like differentiation. The expression of *SPP1* was significantly reduced under differentiating conditions (*p* = 0.0021 for serum and *p* = 0.0236 for BMP4 treated cultures, *n* = 5 experiments). Reduction of *SPP1* expression was associated with the significant downregulation of *NANOG* expression in spheres cultured in the presence of 2% serum and *OCT3/4* in BMP4 -treated spheres (Figure 4E). Treatment with 2% serum or to less extend with BMP4 reduced the expression of stemness genes *NANOG* and *OCT4* to the levels detected in adherent cells (the Supplementary Figure S3). The expression of *TUB-bIII* did not changed significantly at the mRNA level, but we observed the increase of TUB-bIII staining intensity in BMP4 treated cultures indicative of increased protein expression (Figure 4D). These data demonstrate that forced differentiation of GIC cultures is associated with the reduction in the *SPP1* expression.

### Glioma-derived SPP1 supports GIC self-renewal and sphere formation

**Figure 5:**
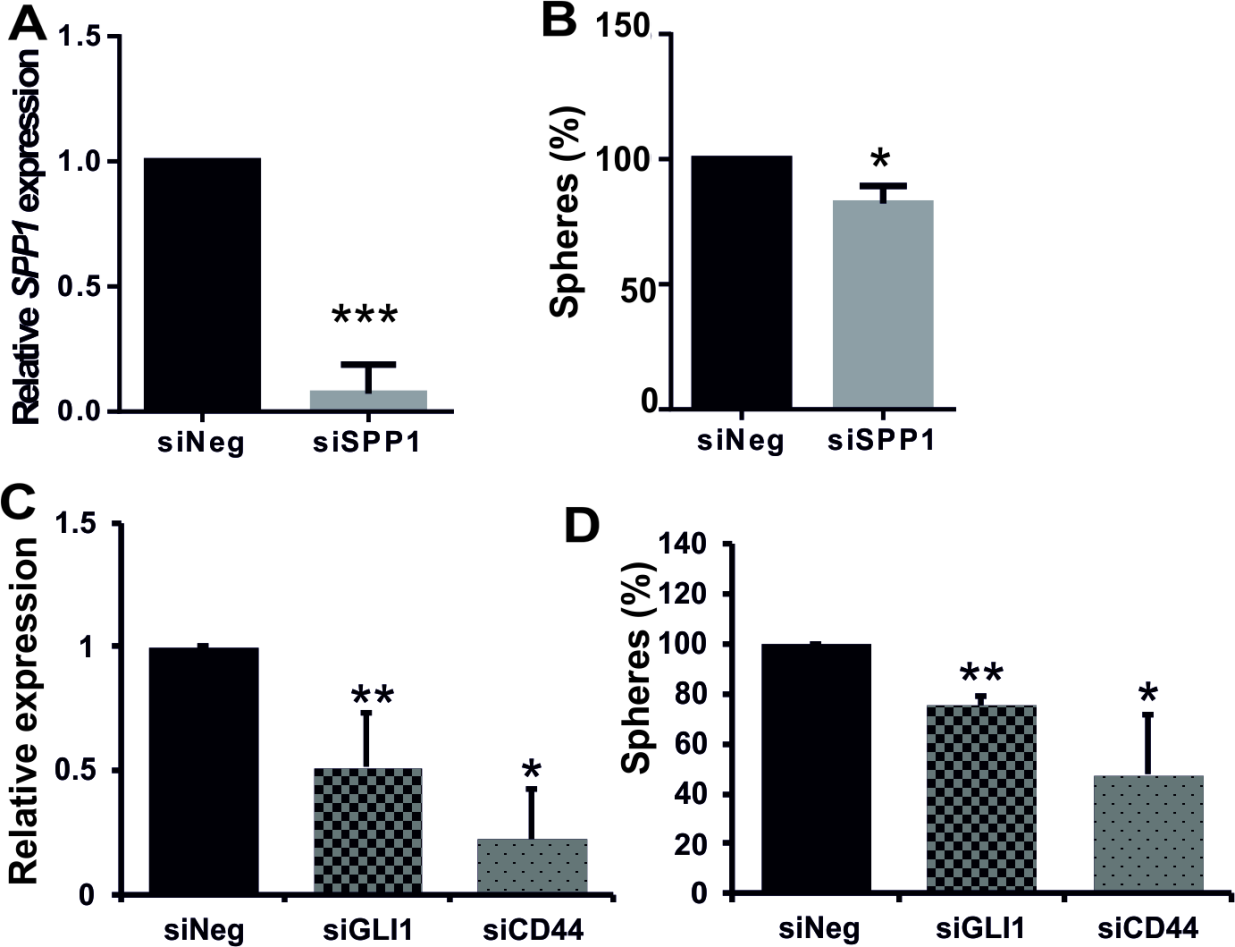
Efficient knockdown of *SPP1* expression in glioma cells reduces sphere formation (**A**–**B**) Knockdown of *SPP1* expression in LN18 cells reduced formation of spheres. Human adherent LN18 glioma cells were transfected with siRNAs ON-Target PlusSMARTpool Human *SPP1* or ON-TARGETplus Non-targeting Pool (Dharmacon) using 4D-nucleofector AMAXA. After 24 h cells were seeded at a low density (4000 cells/ml) onto non-adherent plates and cultured in a defined medium. Resulting spheres (100–200 μm in size) were counted 7 days after transfection (B). In parallel, the expression of *SPP1* in parental cultures was determined by qPCR 48 h after transfection (A) to evaluate efficacy of gene silencing, *n* = 3. (**C**–**D**) Knockdown of *GLI1* or *CD44* expression in LN18 cells impaired sphere formation. LN18 glioma cells were transfected with ON-Target PlusSMARTpool Human *GLI1* or *CD44* or ON-TARGETplus Non-targeting Pool (Dharmacon) siRNAs using 4D-nucleofector AMAXA. The expression of *GLI1* and *CD44* in parental cultures was determined by qPCR 48 h after transfection; data are means ± s.d., *n* = 3 (C). After transfection cells were seeded at a low density onto plates dedicated for a cell suspension culture and cultured 7 days in a defined medium. The resulting spheres (100–200 μm in size) were counted (D). The results are expressed as mean ± s.d.; *P* values were considered significant when *P ≤ 0.05 and **P ≤ 0.01, *n* = 3.

To determine if tumour-derived SPP1 plays a role in glioma sphere formation, we knocked down its expression with the specific siRNA in cells from dissociated spheres and analysed a number of resulting secondary spheres. We demonstrated that siRNA-mediated knockdown of *SPP1* expression in human LN18 cells reduced their ability to form spheres (Figure 5). GIC spheres were counted as spheres if they reached 100–200 μm in size; quantification was performed 7 days after transfection. *SPP1* knockdown in LN18 cells was very efficient (80% reduction of the *SPP1* mRNA level as determined by qPCR) (Figure 5A) and the number of spheres was reduced by 20% (Figure 5B). This moderate reduction could be due to a transitory nature of *SPP1* knockdown during 7 days of sphere culturing. Previous studies suggested the important role of autocrine and paracrine SPP1-CD44 signalling in maintenance of glioma initiating cells [20, 21]. To evaluate the role of GLI1-SPP1-CD44 signalling in a control of sphere formation, we knocked down the expression of *GLI1* or *CD44*. *GLI1* knockdown was verified by qPCR and CD44 knockdown was confirmed by qPCR, Western blotting and flow cytometry (Supplementary Figure S4). The efficient reduction of GLI1 or CD44 expression reduced sphere formation by 22% and 55%, respectively (Figure 5C–5D).

**Figure 6:**
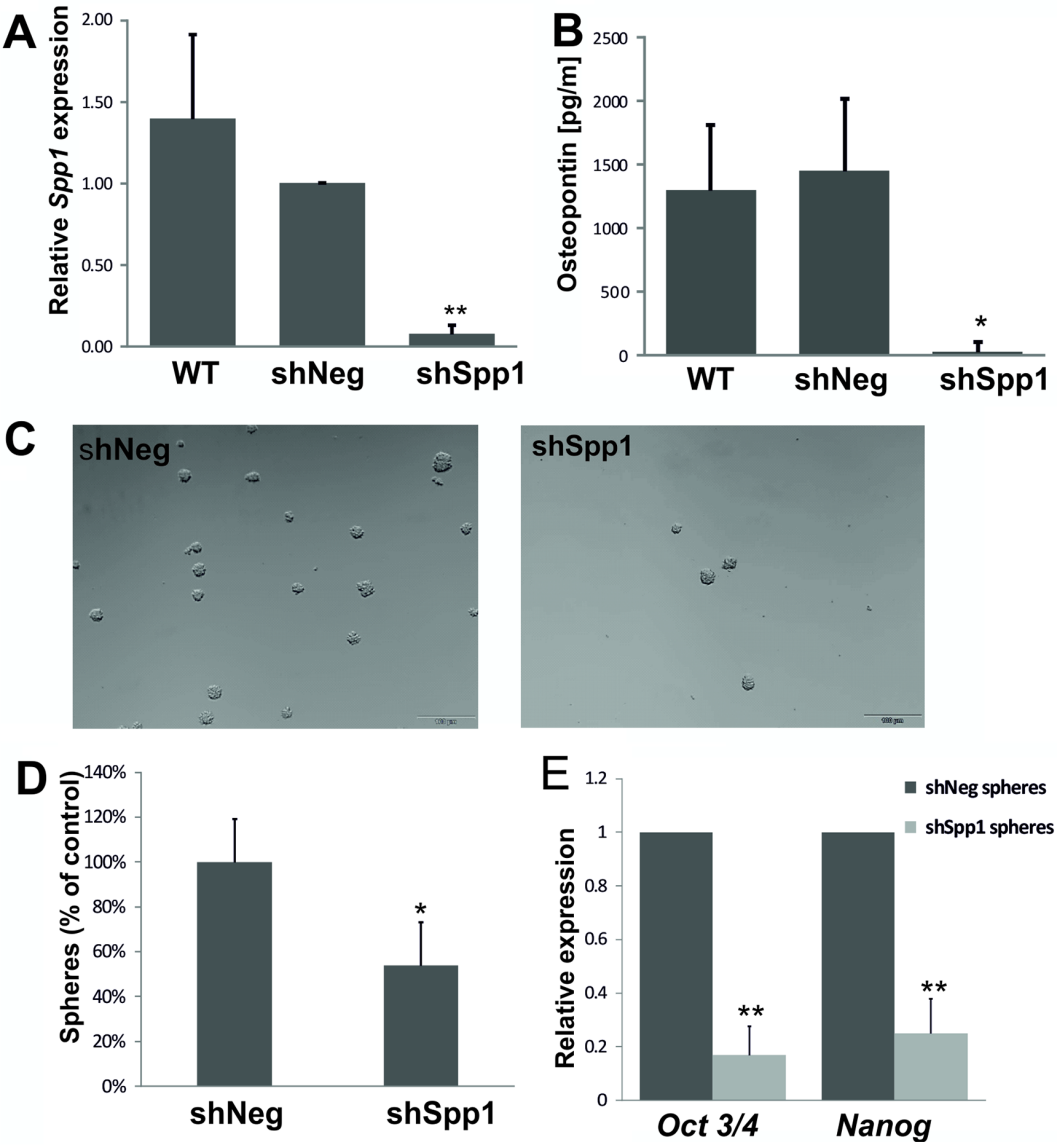
Stable knockdown of *Spp1* expression in C6 glioma cells reduces sphere formation and stemness factor expression (**A**–**B**) Efficient stable knockdown of *Spp1* expression in rat C6 glioma cells was confirmed by qPCR and ELISA. Control shNeg cells expressed *Spp1* and SPP1/osteopontin at the similar level as parental C6 cells (WT). (**C**) Stable knockdown of *Spp1* expression in C6 glioma cells inhibited formation of spheres. Cells were seeded at a low density (4000 cells/ml) onto non-adherent plates and cultured in DMEM/F-12 medium, supplemented with B27, 20 ng/ml bFGF, 20 ng/ml EGF and antibiotics. Representative images show the reduced formation of spheres (100–200 μm in size) derived from the shSpp1 glioma cells 14 days after seeding. (**D**) Quantification of resulting spheres derived from shNeg and shSpp1 glioma cells was performed 14 days after seeding; data are means ± s.d., *n* = 3. (**E**) Reduced expression of *Nanog* and *Oct3/4* in spheres derived from shSpp1 glioma cells. Spheres were collected by centrifugation 14 days after seeding and the expression of *Nanog* and *Oct3/4* in cultures was determined by qPCR; data are presented as means ± s.d., *n* = 3.

To achieve long term knockdown of Spp1, we developed C6 glioma cell lines stably transduced with lentiviral vectors carrying a control shNeg or shSpp1 (corresponding to a siRNA sequence). Transduced clones were sorted by flow cytometry for GFP (encoded in a lentiviral vector) to reduce cell handling before establishment of stably clones. The control shNeg cells expressed the similar level of *Spp1* mRNA and protein as parental C6 glioma cells (Figure 6A, 6B). The levels of *Spp1* mRNA and protein were reduced by > 90% in shSpp1 clones (Figure 6A, 6B). Knockdown of *Spp1* in glioma cells did not affect basal cell viability and proliferation determined with MTT metabolism and BrdU incorporation test (Supplementary Figure S5). However, C6 glioma cells depleted of Spp1 had reduced sphere forming capacity (> 50%) (Figure 6C, 6D) and the reduced expression of stemness factors *Nanog* and *Oct3/4* (Figure 6E).

### The CD44 binding domain of SPP1 contributes to self-renewal of glioma cells

**Figure 7:**
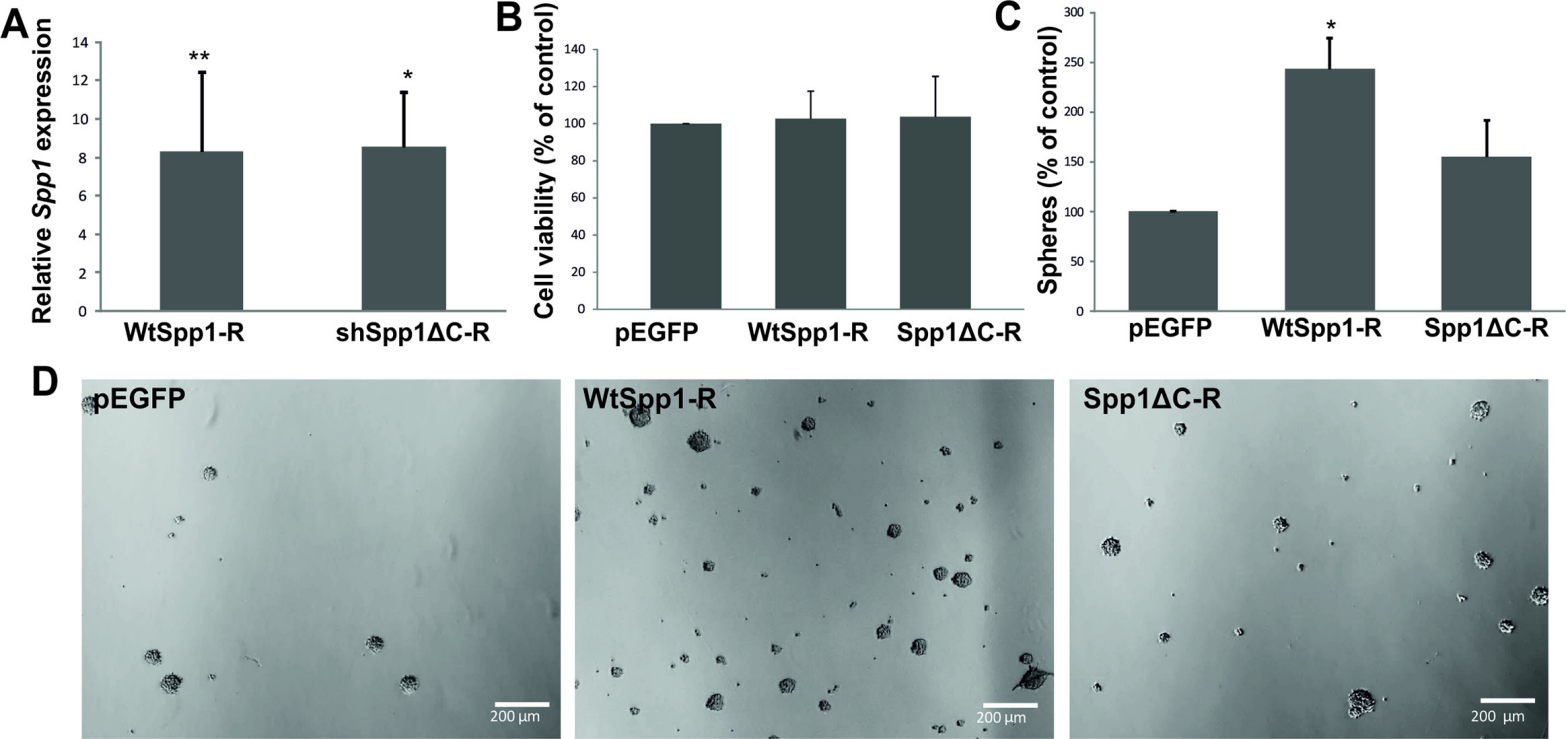
The CD44 binding domain of Spp1 is critical for sphere formation (**A**) C6 glioma cells stably expressing shSpp1 were transfected with various constructs: a control pEGFP, a shRNA resistant, wild type Spp1 (WtSpp1-R) or a shRNA resistant Spp1 lacking a CD44 binding domain (Spp1ΔC-R). Twenty four hours after transfection cells were seeded (8000 cells/ml) under sphere forming conditions (DMEM/F-12 medium with B27, 20 ng/ml bFGF, 20 ng/ml EGF and antibiotics). Reconstitution of *Spp1* expression in cells transfected with WtSpp1-R or Spp1ΔC-R was determined by qPCR in respective cultures and related to values obtained for shSpp1 cells. Data are presented as means ± s.d., *n* = 3. (**B**) Reconstitution of Spp1expression in cells transfected with a control pEGFP, WtSpp1-R or Spp1ΔC-R had no influence cell viability (as determined by MTT metabolism assay 24 h after transfection). (**C**) Only reconstitution of *Spp1* expression in glioma cells transfected with a WtSpp1-R restored cell capacity to form spheres. Data are presented as means ± s.d., *P* values were considered significant when *P ≤ 0.05 and **P ≤ 0.01, *n* = 3. (**D**) Representative images show the reduced formation of spheres derived from the shSpp1 glioma cells transfected with pEGFP 7 days after seeding. Overexpression of the construct coding for a wild type (WtSpp1-R) restored sphere forming capacity of glioma cells; overexpression of Spp1ΔC-R did not restore sphere forming capacity.

A recent study in a murine model of PDGFB-driven gliomas (resembling a proneural type GBM) showed that osteopontin secreted by tumour-associated astrocytes, via CD44 signalling promotes stem cell properties and glioma resistance to radiation [20]. We demonstrate that knockdown of CD44 in glioma cells reduced sphere formation suggesting an important role of CD44-SPP1 interactions in GIC self-renewal. A C-terminus of SPP1 carries a CD44 binding site. To elucidate if the CD44 binding domain of Spp1 is critical for glioma cell capacity to self-renew, we generated constructs carrying RNA interference resistant (R) variants of a wild type Spp1 or a C-terminal truncated Spp1. These constructs were overexpressed in Spp1-depleted glioma cells and the efficient reconstitution of the Spp1 expression was achieved in transfected cells, as determined by qPCR at 48 h after transfection (Figure 7A). Cell proliferation of different clones overexpressing a neutral plasmid pEGFP, WtSpp1-R or Spp1ΔC-R was similar, as determined by a BrdU incorporation assay (Figure 7B). However, the transfected cells differed in their ability to form spheres. GIC spheres were counted 7 days after transfection. Glioma cells depleted of the endogenous Spp1 and overexpressing a neutral plasmid pEGFP failed to form spheres. Overexpression of WtSpp1-R restored glioma cell capability to form spheres, while overexpression of Spp1ΔC-R did not restore a cell ability to form spheres (Figure 7C, 7D).

## DISCUSSION

The main findings of this study are: i) *SPP1* overexpression in glioma initiating cells, ii) a novel mechanism of transcriptional regulation of the *SPP1* by GLI1 and OCT4, and iii) demonstration of the supportive role of tumour-derived osteopontin/SPP1 in self-renewal of glioma initiating cells. We confirmed upregulated expression of a full form of SPP1 - *SPP1-e* in human glioblastomas and several glioma cell lines. We demonstrated that transcription factors OCT4 and GLI1, involved in maintenance of the stemness phenotype in embryonic and cancer stem cells, bind to the *SPP1* gene in glioma cells, but not in normal astrocytes. This binding was detected in human glioma cell lines and GBM patient-derived WG4 cultures. The *GLI1* expression was significantly upregulated in GBMs in comparison to pilocytic astrocytomas and normal brains (Figure 2G). Silencing of *GLI1* expression with siRNA in human glioma cells reduced the levels of *SPP1* expression and protein secretion. Knockdown of *GLI1* significantly reduced sphere formation (Figure 5C, 5D). Binding of GLI1 to the *SPP1* gene promoter was reported in metastatic melanoma cells [26] indicating it could be a general feature of malignant cells. The *OCT4* expression was not significantly different between PA and GBM (Figure 2G). Due to its very low level in glioma cells, we could not demonstrate the efficacy of OCT4 silencing (not shown) and prove its role in the transcriptional SPP1 regulation. However, we demonstrate the up-regulated *OCT4* expression in GIC-enriched cells isolated in the side population and in glioma sphere formation assays. We demonstrated that OCT4 binds to the regulatory element in the first intron of *SPP1* gene in a manner typical for embryonic cells. Regulation of *Spp1* expression by the Oct4 transcription factor, which binds to the first intron of the murine *Spp1* gene, was previously demonstrated in mouse embryonic cells at an early, pre-implantation stage of development [28]. Altogether, our results show a novel mechanism of the transcriptional *SPP1* regulation, which operates in malignant tumour cells containing a subpopulation of rare cells with stemness features.

We demonstrate that even in T98G and LN18 glioblastoma cells exhibiting low levels of *SPP1* expression, the expression of *SPP1* is highly up-regulated in the Rhod123(–) subpopulation and glioma spheres when compared with its levels in adherent cells. Moreover, the increased *SPP1* expression is associated with up-regulated *NANOG* and *OCT3/4* mRNA levels. The higher *SPP1* expression in LN18 spheres versus adherent cells is associated with the significantly increased *GLI1* expression (not shown). This is the first demonstration of concomitant up-regulation of *SPP1* and stemness transcription factors in glioma initiating cells.

In the present study we show the important role of tumour-derived SPP1 in stemness maintenance. We demonstrate that RNAi against *SPP1* reduced the number of spheres formed by LN18 and rat C6 glioma cells, and down-regulated *OCT4* and *NANOG*. Rat C6 glioma cells with stable knockdown of *Spp1* had reduced levels of *Oct3/4* and *Nanog* expression in sphere cultures along with the reduced sphere numbers (Figure 6). Our observation is consistent with recently reported data demonstrating that stably knockdown of *SPP1* in U87MG and GBM4371 glioma cells reduced their sphere forming capacity. The authors showed time-dependent decrease of *OCT3/4*, *NANOG* and *SOX2* expression in glioma spheres after *SPP1* knockdown [21]. In murine PDGFB-induced glioma cultures and primary human GBM cultures stroma-derived osteopontin was responsible for up-regulation of *NANOG*, *SOX2*, *OCT4*, and *ID1* expression [20]. The crucial role of SPP1 in maintenance of human GIC stemness is strengthen by our results demonstrating the reduced *SPP1* expression in LN18 sphere cultures undergoing differentiation in the presence of BMP4 or 2% FBS (Figure 4). This is in agreement with the observations of up-regulated expression of TUJ1 and GFAP, neural differentiation markers, in GBM4371 spheres in response to 10% FBS and withdrawal of growth factors EGF and bFGF [21]. Altogether, these data support the crucial role of ostepontin in maintaining the stemness phenotype of glioma initiating cells across various models. Undoubtedly further research is needed to verify the existence of positive loops in which SPP1 would increase the expression of OCT4 or other stemness factors, which in turn would regulate *SPP1* expression. The role of SPP1 in sphere-forming capacity of cancer stem cells is likely cell type dependent. In mammary cancer spheroids blockade of osteopontin with a specific antibody decreased cell proliferation [36]. In the hematopoietic stem cell niche osteopontin inhibited proliferation of hematopoietic stem cells [37] in a manner consistent with maintaining quiescence [38].

Our results defined mechanisms mediating SPP1 action on glioma cells. We demonstrate the importance of SPP1-CD44 interaction in maintaining the stemness phenotype as a variant of *Spp1* lacking C-terminal domain responsible for interactions with CD44 was not able to restore the impaired sphere formation (Figure 7). Using a rescue strategy, we demonstrate that overexpression of WtSpp1-R, but not Spp1ΔC-R, restored glioma cell capability to form spheres (Figure 7C, 7D). This provides evidence that the C-terminus of SPP1 carrying the CD44 binding site is critical for stemness maintaining SPP1 activity. The variant of Spp1 lacking C-terminal domain contains an intact RGD domain responsible for integrin binding. The lack of functional rescue suggests that binding of Spp1 with integrins was not sufficient to restore sphere formation capacity. CD44, a transmembrane glycoprotein, was initially identified as a receptor for hyaluronic acid, and high CD44 expression was associated with cancer stem cells and epithelial-mesenchymal transition. Previous studies have shown that CD44 expression is upregulated in GIC and the CD44^high^ cells have higher self-renewal capacity and generate more spheres than CD44^low^ population [39]. We detected similar levels of CD44 mRNA and protein in adherent and spheres cultures of LN18 cells. Forced differentiation did not change CD44 expression levels (Supplementary Figure 4A). In murine model of PDGF-induced glioma osteopontin secreted by glioma-associated astrocytes enhanced the cancer stem cell phenotype through interactions with CD44 [20]. Transcriptomic data showed that *Spp1* was up-regulated specifically in glioma-associated astrocytes compared to normal astrocytes in murine PDGFB-driven gliomas and proneural human GBMs [40]. Our data show an alternative mechanism of an autocrine action of glioma-derived SPP1/osteopontin. Despite different cell sources of SPP1/osteopontin (whether SPP1 is produced by tumour or stromal cells) all presented data confirmed the important role of osteopontin-CD44 interactions in maintaining the glioma stem cell phenotype. Our data show a novel transcriptional mechanism driving *SPP1* expression in glioma cells that is not in operation in non-transformed astrocytes.

## MATERIALS AND METHODS

### GBM and PA samples

Frozen glioma biopsies were obtained from the Brain Tumour Tissue Bank (London Health Sciences Centre, CA) and Institute of Psychiatry and Neurology (IPIN) in Warsaw. Additional pilocytic astrocytoma (PA) samples and 5 normal brain tissues were obtained the Children's Memorial Health Institute, Warsaw. The study was conducted under protocol #14/KBE/2012, approved by the Research Ethics Board at IPIN.

### Cell culture

Rat C6 and human T98G, LN18, LN229, U87 MG, U373 glioma cells, human teratocarcinoma Ntera and HeLa cells were from ATCC. Cells were passaged twice a week up to a passage 20 and were maintained for no longer than 15 weeks. Human cells were grown in DMEM with 10% foetal bovine serum (FBS) and antibiotics (50 U/ml penicillin, 50 μg/ml streptomycin); C6 cells were grown in DMEM with 10% newborn calf serum (NCS) and antibiotics as described [41]. Normal human astrocytes were from Lonza and were cultured in the supplier-recommended medium (Lonza, CC-3186) for 2–3 weeks before experiments. Astrocyte cultures were > 98% GFAP-positive and were cultivated in DMEM with 10% FBS, passaged every week. WG4 primary glioma cultures originated from a GBM patient surgical sample. Freshly resected tumour tissue was washed in Hank's balanced sodium solution (HBSS) and subjected to mechanical and enzymatic dissociation using Neural Tissue Dissociation Kit (MiltenyiBiotec) according to the manufacturer's instructions. Tumour cells were re-suspended in DMEM F-12 supplemented with 10% FBS (Gibco) and plated at a density of 1–2 × 10^6^ cells/60 mm plate. The fresh medium was replaced every 4 days.

### Chromatin immunoprecipitation (ChIP)

ChIP assays were performed on human glioma cells and non-transformed astrocytes as previously described [42, 43]. ChIP was performed with Chip-IT Express Active Motif kit according to manufacturers’ protocol. Cells were fixed with 2% formaldehyde, sonicated with Bioruptor Plus (Diagenode) with 9 cycles 30 sec ON/OFF. Positive controls for ChIP were from Active Motif Chip-IT Control Kit Human (#53010). Antibodies for ChIP included: anti-GLI1 (Santa Cruz N-16x), anti-OCT4 (Abcam ab18976), goat IgG (Abcam ab37373), rabbit IgG (Calbiochem NI01); all antibodies were used at 3 μg per reaction. The following ChIP-PCR primers were used: for the *OCT4* binding site in the first intron of *SPP1* gene forward CACCTAAGTAGCACCTACTTG, reversed CCTACAAACAGATCAACAGTAAC and for the *GLI1* binding site in the *SPP1* gene promoter: forward CTGACAGAAAATCCTACTCAGAAAA, reversed: AAA GTAGGAAATGGATGCTGCG. Two putative GLI1 binding sites have been identified in the *SPP1* promoter using MathInspector (Genomatix, Germany) and the Ensembl funcgen database as described [27]. ChIP primers were designed for both putative sites but only the binding to the proximal GLI binding site of the *SPP1* gene was confirmed. Primers for the potential OCT4 binding site in the first intron of the *SPP1* gene corresponded to the Oct4 binding site in the murine *Spp1* gene previously described [28]. The PCR products were resolved in 1% agarose gel with ethidium bromide and visualized with UV light.

### Isolation of glioma initiating cells (GIC) by flow cytometry

For side population isolation, Rhodamine 123 (Rhod123) exclusion assay was performed. Glioma cells from the earliest passages were trypsinized and 10^7^ cells were incubated with 0.1 μg/ml Rhod123 (in DMSO, Sigma) for 20 min in 37°C. Cells were rinsed, re-suspended in 0.5 ml of medium and placed in 37°C for 90 min for Rhod123 exclusion. A portion of cells kept on ice to inhibit the exclusion of Rhod123 served as a positive control. Fractions of Rhod123(+) and (–) cells were sorted using FACS Aria (Becton Dickinson, USA) and collected in Qiagen RLT lysis buffer. We did not observe any significant increase in the number of dead cells in our preparation, cell debris were removed from quantification by appropriate gating as previously described [32]. For the experiment presented in the Supplementary Figure S3B, prior to a flow cytometry analysis the cells were labelled with 5 μg/ml of propidium iodide to determine numbers of live/dead cells.

### Sphere cultures and differentiation induction

For sphere forming assay, cells were seeded at a low density (4000 or 8000 cells/ml of medium) onto non-adherent plates and cultured in DMEM/F-12 medium (Gibco, 31331-028), supplemented with B27 (Gibco, 0080085SA), 20 ng/ml bFGF (Miltenyi Biotec), 20 ng/ml EGF (Stemcell Technologies) and antibiotics. After 7 or 14 days resulting spheres were collected by centrifugation at 1500 rpm and lysed in Qiagen RLT lysis buffer.

For differentiation experiments, spheres were maintained for 8 days in the sphere culture medium followed by incubation for 5 days in the presence of 100 ng/ml BMP4 (Sigma-Aldrich, Germany) or 2% FBS.

### Immunoblotting and immunofluorescence

Western blot analysis was performed as previously described [44]. Primary antibodies included: anti-SOX2, anti-OCT4A (Cell Signaling Technology, Beverly MA, USA), anti-NANOG (Cell Signaling Technology, Beverly MA, USA). Immunocomplexes were visualized using ECL (Amersham, Germany). The membranes were stripped and re-probed with horseradish peroxidase-conjugated anti-β-actin antibody (1:10000, #A3854 Sigma-Aldrich, Saint Louis, MO, USA). The molecular weight of proteins was estimated with pre-stained protein markers (Sigma-Aldrich, Saint Louis, MO, USA). CD44 was detected by Western blotting with polyclonal Sheep IgG anti-human CD44 (#AF3660, R&D System, Minneapolis, MN, USA, 1:500) followed by a rabbit Anti-Sheep IgG-HRP conjugate (#AP147P, Merck Millipore KGaA, Darmstadt, Germany, 1:5000). For flow cytometry we used Alexa Fluor 700 Mouse Anti-Human CD44 Clone G44-26 RUO (#561289, BD Pharmingen); Alexa Fluor 700 Mouse IgG2b, κ Isotype Control RUO (#560543, BD Pharmingen).

For immunofluorescence analysis, cells grown on coverslips were fixed with 4% paraformaldehyde (PFA). After blocking for 1 h with 0.1% Triton X-100 containing 1% bovine serum albumin, sections were incubated with anti-β Tubulin III antibody (Millipore, *Temecula, CA, USA*) diluted in 1% bovine serum albumin containing 0.1% Triton X-100 at 4°C overnight. Reactions were visualized by incubating with a secondary, anti-mouse antibody conjugated to Alexa-555 (Life Technologies, Karlsruhe Germany). Cells were counterstained with 1 μg/ml DAPI and images were acquired with Olympus X70 fluorescent microscope.

### Real- time PCR

Total RNA was isolated from glioma cells using Qiagen RNeasy kit, 1 μg was used as a template. Amplifications were performed in duplicates in 20 μl containing 2× SYBR PCR MasterMix (Applied Biosystems) and a set of primers (human *SPP1, NANOG* and *POU5F1_1_SG/OCT4* primers were from QuantiTect Primer Assays: QT01008798, QT01844808 and QT00210840, respectively, Qiagen). Sequences of primers for rat *Spp1* stemness and differentiation genes are in the Table 1. The amount of target mRNA was normalized to the 18S rRNA amplified from the same sample. Data were analysed using the Relative Quantification (^ΔΔ^Ct) method with 7500 System SDS software (Applied Biosystems).

**Table 1:** Sequences of qPCR primers

Gene name	Primers
human *SPP1*	f- TTTGTTGTAAAGCTGCTTTTCCTC r- GAATTGCAGTGATTTGCTTTTGC
human *SPP1-a*	f- AAGCAGCTTTACAACAAATACCCA r- TACTTGGAAGGGTCTGTGGGG
human *SPP1-b*	f- TTGGAAGGGTCTGTGGGGCTAGG r- CCTCCTAGGCATCACCTGTGCCAT
human *SPP1-c*	f- GAATTGCAGTGATTTGCTTTTGC r- AGGACACAGCATTCTGCTTTTC
human *SPP1-d*	f- GAATTGCAGTGATTTGCTTTTGC r- GGAAGGGTCTGCTTTTCCTCA
human *SPP1-e*	f- GAATTGCAGTGATTTGCTTTTGC r- AGGTACATCTTTAGTGCTGCTTTTC
human *GAPDH*	f- AGGGCTGCTTTTAACTCTGGT r- CCCCACTTGATTTTGGAGGGA
human *GLI1*	f- GCCCAGCTTGTCCCACACCG r- AGGAGCGGCGGCTGACAGTA
human *CD44*	f- CCATCTGTGCAGCAAACAACA r- TTCAGGTGGAGCTGAAGCATT
human *GFAP*	f- TCCTGGAACAGCAAAACAAG r- CAGCCTCAGGTTGGTTTCAT
human *SOX2*	f- GGGGAAAGTAGTTTGCTGCC r- CGCCGCCGATGATTGTTATT
human TUBULIN-*βIII*	f- GTACGTGCCTCGAGCCATTCT r- CGTGTAGTGACCCTTGGCCC
18S *rRNA* human/rat	f- AACGAACGAGACTCTGGCATG r- CGGACATCTAAGGGCATCACA
rat *Spp1*	f- TTGTTTCTCAGTTCAGTGGATACATG r- CAGTGGTGTCTGCATGAAAC
rat *Nanog*	f- CCCTTGCCGTTGGGCTGACA r- AAGGCGGAGGAGAGGCAGTCT
rat *Oct3/4*	f- CCCAGCGCCGTGAAGTTGGA r- AGAACGCCCAGGGTGAGCCC

### siRNA experiments and transfection

LN18 cultures were transfected with 25 nM SMARTpool: siGENOME SPP1 siRNA and siGENOME Non-Targeting siRNA Pool #1 (Thermo Scientific) using DharmaFECT. Transfection efficacy estimated with a rhodamine labelled control siRNA was 89%. Osteopontin levels in glioma conditioned media were determined by ELISA (#ADI-900-142, ENZO Life Sciences). Human *GLI1* was knocked-down using ON-Target PlusSMARTpool Human GLI1 (L-003896-00-005, Dharmacon), ON-Target PlusSMARTpool Human CD44 siRNA (L-009999-00-0005, Dharmacon); ON-TARGETplus Non-targeting Pool (D-001810-10-05) served as a control. Transfection was performed with 4D-nucleofector AMAXA (Lonza) using 1.5 μg of siRNA/1 × 10^6^ cells. Efficacy of transfection was verified after 48 h.

### *Spp1* knock-down with a lentiviral shRNA

Oligonucleotides corresponding to Spp1 shRNA (5′-AGCTAGTCCTAGACCCTAA-3′ against rat *Spp1*, NM_012881.2) and control shRNA Neg (5′ GTCTCCACG CGCAGTACATTT-3′, based on pSilencer shNeg from Ambion) were cloned into a lentivector pLenti-U6-shRNA-Rsv(GFP-Puro) (GenTarget Inc, San Diego, USA). Rat C6 glioma cells (5 × 10^4^) were seeded onto 24-well plates, transduced with 5 × 10^5^ ready-to-use lenitiviral particles in 0.5 ml culture medium according to a manufacturer's protocol, and shSpp1 or shNeg clones were enriched by incubation with puromycin 2 μg/ml (BioShop, Canada Inc., Burlington). Efficacy of *Spp1* gene silencing in heterogeneous pools was examined by qPCR and ELISA. For “rescue” experiments 6 × 10^4^ C6 LVSpp1 cells were seeded on 24-wells plates and after 24 h cells were transfected with 18 ng/μl of plasmid DNA and a transfection reagent Viromer^®^ RED (Lypocalyx, Germany). Twenty four hours after transfection cells were transferred to sphere culture medium and grown for 7 or 14 days.

### Generation of shRNA resistant Spp1 plasmids for rescue experiments

In order to generate shRNA resistant constructs coding for a wild type and a ΔC-term pSpp1 (lacking a CD44 domain of Spp1), we performed a site-directed mutagenesis. We designed mismatched oligonucleotides binding to Spp1 shRNA binding site: Spp_mut_R: CTTCCTTACTCTTTGGATCGA GTACTAGTTTGTCC TCATGGCTGTG and Spp_mut_F: CCATGAGGACAAA CTAGTACT CGATCCAAAGAGTAAGGAAGATGAT AG. Primer-extension reaction amplified pEGFP-N1 vector carrying a wild type *Spp1* cDNA (wtSpp1) or *Spp1ΔC* cDNA. The PCR reaction was performed with Phusion polymerase (New England Biolobs) using reaction conditions recommended by the manufacturer. Primer annealing was performed in 60°C. In order to eliminate template DNA, PCR product was digested with DpnI restriction enzyme and further transformed into XL1-Blue E.coli strain. Designed mutagenesis introduced point mutations without affecting aminoacid sequence and in the same time generated a new restriction site for SpeI enzyme. Simultaneous digestion with SpeI (Thermo Scientific) and EcoRI (Thermo Scientific) helped us to preselect proper clones before sequencing. The resulting plasmids were verified by sequencing.

### Statistical analysis

Statistical significance was determined by Student's *t-test*; *p value*s < 0.05 were considered significant. The results are expressed as a mean ± standard deviation from 3–5 independent experiments (different cell passages).

## SUPPLEMENTARY MATERIALS FIGURES AND TABLES



## References

[1] Anborgh PH, Mutrie JC, Tuck AB, Chambers AF (2010). Role of the metastasis-promoting protein osteopontin in the tumour microenvironment. J Cell Mol Med.

[2] Brown LF, Papadopoulos-Sergiou A, Berse B, Manseau EJ, Tognazzi K, Perruzzi CA, Dvorak HF, Senger DR (1994). Osteopontin expression and distribution in human carcinomas. Am J Pathol.

[3] Tuck AB, Chambers AF, Allan AL (2007). Osteopontin overexpression in breast cancer: knowledge gained and possible implications for clinical management. J Cell Biochem.

[4] Rittling SR, Chambers AF (2004). Role of osteopontin in tumour progression. Br J Cancer.

[5] Bellahcène A, Castronovo V, Ogbureke KU, Fisher LW, Fedarko NS (2008). Small integrin–binding ligand N–linked glycoproteins (SIBLINGs): multifunctional proteins in cancer. Nat Rev Cancer.

[6] McAllister SS, Gifford AM, Greiner AL, Kelleher SP, Saelzler MP, Ince TA, Reinhardt F, Harris LN, Hylander BL, Repasky EA, Weinberg RA (2008). Systemic endocrine instigation of indolent tumor growth requires osteopontin. Cell.

[7] Scatena M, Liaw L, Giachelli CM (2007). Osteopontin: a multifunctional molecule regulating chronic inflammation and vascular disease. Arterioscler Thromb Vasc Biol.

[8] Cho HJ, Kim HS (2009). Osteopontin: a multifunctional protein at the crossroads of inflammation, atherosclerosis, and vascular calcification. Curr Atheroscler Rep.

[9] Sharif SA, Du X, Myles T, Song JJ, Price E, Lee DM, Goodman SB, Nagashima M, Morser J, Robinson WH, Leung LL (2009). Thrombin-activatable carboxypeptidase B cleavage of osteopontin regulates neutrophil survival and synoviocyte binding in rheumatoid arthritis. Arthritis Rheum.

[10] Shao Z, Morser J, Leung LL (2014). Thrombin Cleavage of Osteopontin Disrupts a Pro-chemotactic Sequence for Dendritic Cells, which is Compensated by the Release of its Pro-chemotactic C-Terminal Fragment. J Biol Chem.

[11] Yamaguchi Y, Shao Z, Sharif S, Du XY, Myles T, Merchant M, Harsh G, Glantz M, Recht L, Morser J, Leung LL (2013). Thrombin-cleaved fragments of osteopontin are overexpressed in malignant glial tumors and provide a molecular niche with survival advantage. J Biol Chem.

[12] Weller M, Cloughesy T, Perry JR, Wick W (2013). Standards of care for treatment of recurrent glioblastoma--are we there yet?. Neuro Oncol.

[13] Saitoh Y, Kuratsu J, Takeshima H, Yamamoto S, Ushio Y (1995). Expression of osteopontin in human glioma. Its correlation with the malignancy. Lab Invest.

[14] Sreekanthreddy P, Srinivasan H, Kumar DM, Nijaguna MB, Sridevi S, Vrinda M, Arivazhagan A, Balasubramaniam A, Hegde AS, Chandramouli BA, Santosh V, Rao MR, Kondaiah P (2010). Identification of potential serum biomarkers of glioblastoma: serum osteopontin levels correlate with poor prognosis. Cancer Epidemiol Biomarkers Prev.

[15] Toy H, Yavas O, Eren O, Genc M, Yavas C (2009). Correlation between osteopontin protein expression and histological grade of astrocytomas. Pathol Oncol Res.

[16] Yan W, Qian C, Zhao P, Zhang J, Shi L, Qian J, Liu N, Fu Z, Kang C, Pu P, You Y (2010). Expression pattern of osteopontin splice variants and its functions on cell apoptosis and invasion in glioma cells. Neuro Oncol.

[17] Jan HJ, Lee CC, Shih YL, Hueng DY, Ma HI, Lai JH, Wei HW, Lee HM (2010). Osteopontin regulates human glioma cell invasiveness and tumor growth in mice. Neuro Oncol.

[18] Singh SK, Hawkins C, Clarke ID, Squire JA, Bayani J, Hide T, Henkelman RM, Cusimano MD, Dirks PB (2004). Identification of human brain tumour initiating cells. Nature.

[19] Piccirillo SG, Binda E, Fiocco R, Vescovi AL, Shah K (2009). Brain cancer stem cells. J Mol Med (Berl).

[20] Pietras A, Katz AM, Ekström EJ, Wee B, Halliday JJ, Pitter KL, Werbeck JL, Amankulor NM, Huse JT, Holland EC (2014). Osteopontin-CD44 signaling in the glioma perivascular niche enhances cancer stem cell phenotypes and promotes aggressive tumor growth. Cell Stem Cell.

[21] Lamour V, Henry A, Kroonen J, Nokin MJ, von Marschall Z, Fisher LW, Chau TL, Chariot A, Sanson M, Delattre JY, Turtoi A, Peulen O, Rogister B (2015). Targeting osteopontin suppresses glioblastoma stem-like cell character and tumorigenicity in vivo. Int J Cancer.

[22] Wang D, Yamamoto S, Hijiya N, Benveniste EN, Gladson CL (2000). Transcriptional regulation of the human osteopontin promoter: functional analysis and DNA-protein interactions. Oncogene.

[23] Wai PY, Mi Z, Gao C, Guo H, Marroquin C, Kuo PC (2006). Ets-1 and runx2 regulate transcription of a metastatic gene, osteopontin, in murine colorectal cancer cells. J Biol Chem.

[24] Schultz J, Lorenz P, Ibrahim SM, Kundt G, Gross G, Kunz M (2009). The functional -443T/C osteopontin promoter polymorphism influences osteopontin gene expression in melanoma cells via binding of c-Myb transcription factor. Mol Carcinog.

[25] Liu YN, Kang BB, Chen JH (2004). Transcriptional regulation of human osteopontin promoter by C/EBPalpha and AML-1 in metastatic cancer cells. Oncogene.

[26] Das S, Harris LG, Metge BJ, Liu S, Riker AI, Samant RS, Shevde LA (2009). The hedgehog pathway transcription factor GLI1 promotes malignant behavior of cancer cells by up-regulating osteopontin. J Biol Chem.

[27] Krystkowiak I, Lenart J, Debski K, Kuterba P, Petas M, Kaminska B, Dabrowski M (2013). Nencki Genomics Database--Ensembl funcgen enhanced with intersections, user data and genome-wide TFBS motifs. Database (Oxford).

[28] Botquin V, Hess H, Fuhrmann G, Anastassiadis C, Gross MK, Vriend G, Schöler HR (1998). New POU dimer configuration mediates antagonistic control of an osteopontin preimplantation enhancer by Oct-4 and Sox-2. Genes Dev.

[29] Hsieh A, Ellsworth R, Hsieh D (2011). Hedgehog/GLI1 regulates IGF dependent malignant behaviors in glioma stem cells. J Cell Physiol.

[30] Jacques TS, Swales A, Brzozowski MJ, Henriquez NV, Linehan JM, Mirzadeh Z, C O’ Malley, Naumann H, Alvarez-Buylla A, Brandner S (2010). Combinations of genetic mutations in the adult neural stem cell compartment determine brain tumour phenotypes. EMBO J.

[31] S Alcantara Llaguno, Chen J, Kwon CH, Jackson EL, Li Y, Burns DK, Alvarez-Buylla A, Parada LF (2009). Malignant astrocytomas originate from neural stem/progenitor cells in a somatic tumor suppressor mouse model. Cancer Cell.

[32] Touil Y, Zuliani T, Wolowczuk I, Kuranda K, Prochazkova J, Andrieux J, Le Roy H, Mortier L, Vandomme J, Jouy N, Masselot B, Segard P, Quesnel B (2013). The PI3K/AKT signaling pathway controls the quiescence of the low-Rhodamine123-retention cell compartment enriched for melanoma stem cell activity. Stem Cells.

[33] Al-Hajj M, Becker MW, Wicha M, Weissman I, Clarke MF (2004). Therapeutic implications of cancer stem cells. Curr Opin Genet Dev.

[34] Chiou SH, Yu CC, Huang CY, Lin SC, Liu CJ, Tsai TH, Chou SH, Chien CS, Ku HH, Lo JF (2008). Positive correlations of Oct-4 and Nanog in oral cancer stem-like cells and high-grade oral squamous cell carcinoma. Clin Cancer Res.

[35] Singh SK, Clarke ID, Terasaki M, Bonn VE, Hawkins C, Squire J, Dirks PB (2003). Identification of a cancer stem cell in human brain tumors. Cancer Res.

[36] Shevde LA, Metge BJ, Mitra A, Xi Y, Ju J, King JA, Samant RS (2010). Spheroid-forming subpopulation of breast cancer cells demonstrates vasculogenic mimicry via hsa-miR-299–5p regulated de novo expression of osteopontin. J Cell Mol Med.

[37] Stier S, Ko Y, Forkert R, Lutz C, Neuhaus T, Grünewald E, Cheng T, Dombkowski D, Calvi LM, Rittling SR, Scadden DT (2005). Osteopontin is a hematopoietic stem cell niche component that negatively regulates stem cell pool size. J Exp Med.

[38] Nilsson SK, Johnston HM, Whitty GA, Williams B, Webb RJ, Denhardt DT, Bertoncello I, Bendall LJ, Simmons PJ, Haylock DN (2005). Osteopontin, a key component of the hematopoietic stem cell niche and regulator of primitive hematopoietic progenitor cells. Blood.

[39] Anido J, Sáez-Borderías A, Gonzàlez-Juncà A, Rodón L, Folch G, Carmona MA, Prieto-Sánchez RM, Barba I, Martínez-Sáez E, Prudkin L, Cuartas I, Raventós C, Martínez-Ricarte F (2010). TGF-β Receptor Inhibitors Target the CD44(high)/Id1(high) Glioma-Initiating Cell Population in Human Glioblastoma. Cancer Cell.

[40] Katz AM, Amankulor NM, Pitter K, Helmy K, Squatrito M, Holland EC (2012). Astrocyte-specific expression patterns associated with the PDGF-induced glioma microenvironment. PLoS One.

[41] Ciechomska IA, Gabrusiewicz K, Szczepankiewicz AA, Kaminska B (2013). Endoplasmic reticulum stress triggers autophagy in malignant glioma cells undergoing cyclosporine a-induced cell death. Oncogene.

[42] Przanowski P, Dabrowski M, Ellert-Miklaszewska A, Kloss M, Mieczkowski J, Kaza B, Ronowicz A, Hu F, Piotrowski A, Kettenmann H, Komorowski J, Kaminska B (2014). The signal transducers Stat1 and Stat3 and their novel target Jmjd3 drive the expression of inflammatory genes in microglia. J Mol Med (Berl).

[43] Kruczyk M, Przanowski P, Dabrowski M, Swiatek-Machado K, Mieczkowski J, Wallerman O, Ronowicz A, Piotrowski A, Wadelius C, Kaminska B, Komorowski J (2014). Integration of genome-wide of Stat3 binding and epigenetic modification mapping with transcriptome reveals novel Stat3 target genes in glioma cells. Biochim Biophys Acta.

[44] Ellert-Miklaszewska A, Dabrowski M, Lipko M, Sliwa M, Maleszewska M, Kaminska B (2013). Molecular definition of the pro-tumorigenic phenotype of glioma-activated microglia. Glia.

