# Supplementary material for: The embryonic type of SPP1 transcriptional regulation is re-activated in glioblastoma
Source: Oncotarget. 2016 Dec 22;8(10):16340–55. doi: 10.18632/oncotarget.14092 (PMC5369967; doi:10.18632/oncotarget.14092)
Supplement: Supplementary file 1 [file oncotarget-08-16340-s001.pdf]

The embryonic type of *SPP1* transcriptional regulation is re-activated in glioblastoma

Supplementary Materials

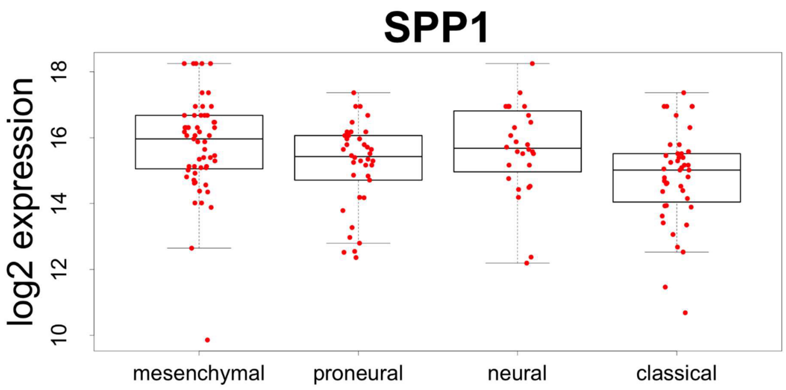

Supplementary Figure S1: The expression of *SPP1* in subtypes of GBM based on TCGA dataset. *SPP1* mRNA expression levels was detected in 528 clinical GBM specimens obtained by TCGA. The value represents log 2 of gene expression value. *SPP1* mRNA levels were not significantly different in subtypes of GBM.

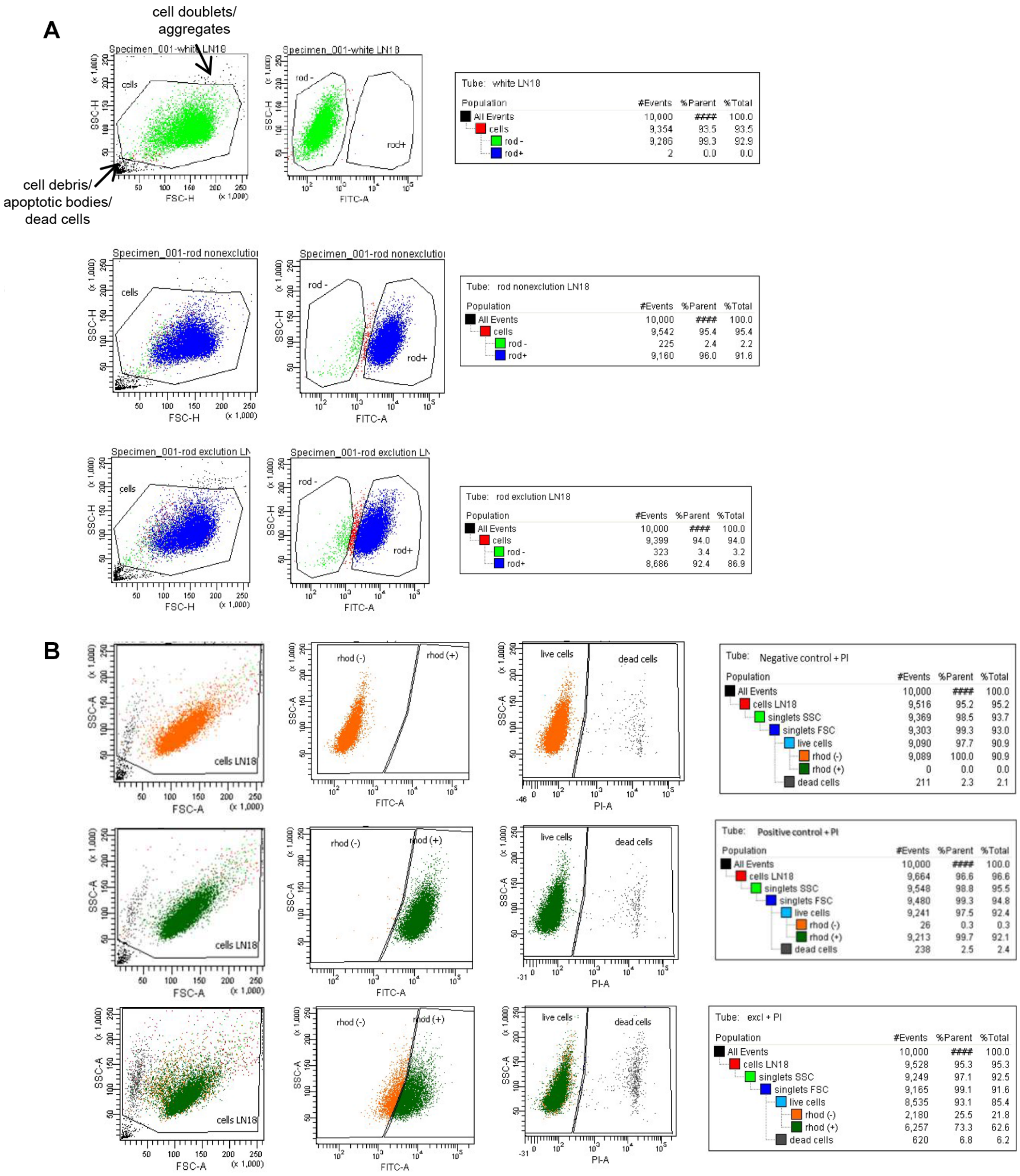

Supplementary Figure S2: (**A**) Gating strategy without propidium iodide. First, the elimination of cell debris, apoptotic bodies, cell doublets, aggregates and dead cells. Then, gating unstained cells (Rhod(–) cells) as a negative control. Cells were stained for 20 min at 37C with 0.1 mM Rhod123. Then gating identication of Rhod (+) cells, as a positive control. After staining, cells were left for 90 min for dye exclusion, then sorting Rhod(+) and Rhod(–) cells. (**B**) Gating strategy with propidium iodide.

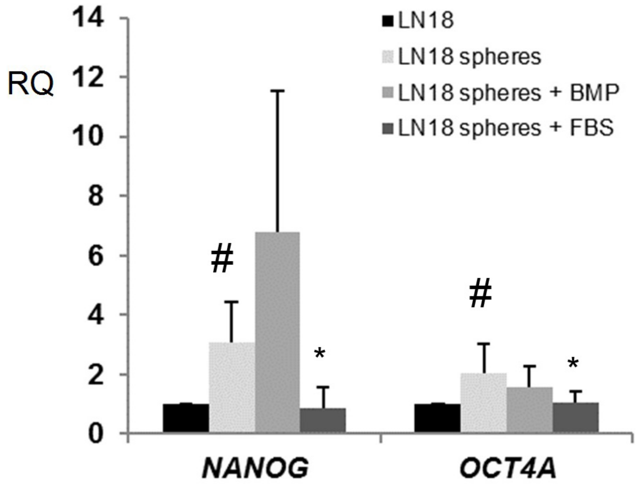

Supplementary Figure S3: BMP4 and 2% serum reduced the expression of stemness genes *NANOG* and *OCT4* to the levels detected in adherent cells. The expression was detected by qPCR and normalized to its levels in adherent cells ($n = 3$); 18S rRNA was used as an internal reference gene.

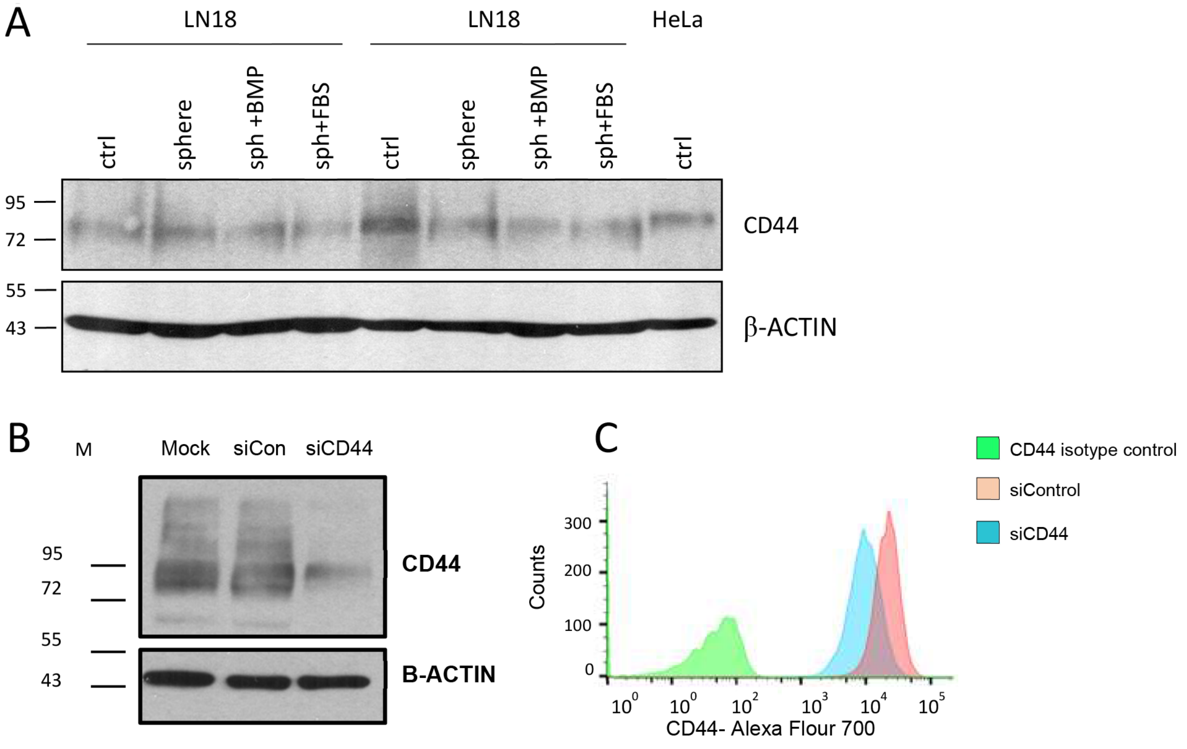

Supplementary Figure S4: The expression of CD44 in glioma adherent and sphere cultures, and the effectiveness of its knockdown in LN18 glioma cells. (**A**) Representative immunoblot shows the CD44 levels in control, adherent cells, in sphere cultures untreated or treated with differentiating factors (BMP4 or FBS) in two experiments; HeLa cells were used as a controls. (**B**) CD44 levels in LN18 cells mock transfected or transfected with sicontrol or siCD44; cells were collected 48 h after transfection. (**C**) Representative graphs showing CD44 expression in glioma cells transfected with siRNA control or siiCD44 as determined by flow cytometry; staining with isotype control antibody in green.

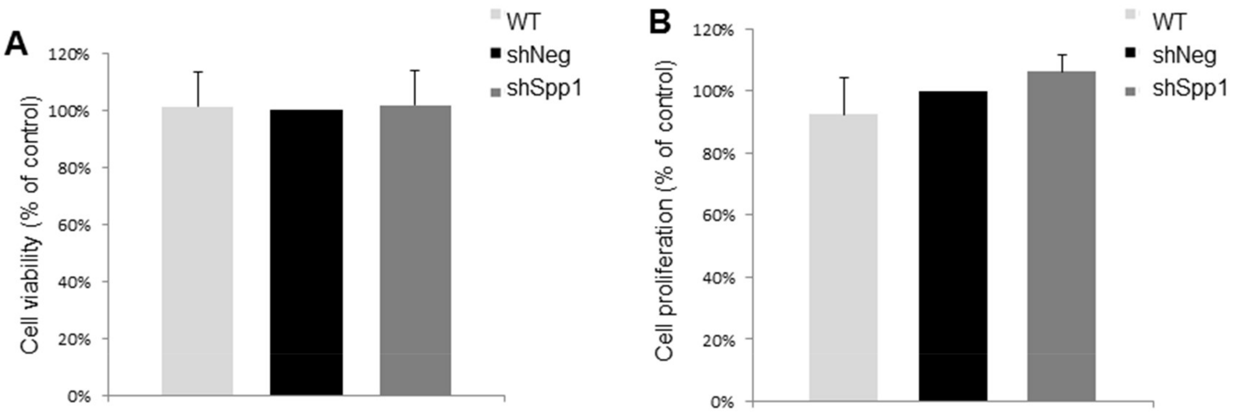

Supplementary Figure S5: Knockdown of Spp1 in glioma cells did not affect basal cell viability (MTT metabolism test) and proliferation (BrdU incorporation test). The effects were determined in parental C6 cells, shNeg and shSpp1 cells. Data were related to shNeg cells and represent means ± s.d. from three experiments, each in triplicates.
